# A letter to the doctor of tomorrow

**DOI:** 10.11604/pamj.2016.25.255.11319

**Published:** 2016-12-21

**Authors:** Luchuo Engelbert Bain

**Affiliations:** 1 Athena Institute for Research on Innovation and Communication in Health and Life Sciences Faculty of Earth and Life Sciences, Vrije Universiteit, Amsterdam, Netherlands

**Keywords:** Doctors, future, medicine, practice

## Editorial

Dear future doctor, Medical practice is an exciting, honorable, respected and morally charged profession with enormous responsibilities and challenges. The art of medicine is three fold: treatment, prevention of disease and palliation. You will be dealing with patients not cases. You shall be called a respected doctor if and only if you treat your patient, not case, with due respect and uphold at all times his human dignity. Always ask yourself, if I am sick, what I shall expect as attitude from my attending physician. Never forget that some diseases are incurable and you will not have much to offer at times. It is time now more than ever before to revise and get updates on your palliative care lectures. Your time, empathy and respect towards the terminally ill is an honorable responsibility. Prevention is surer and cheaper than cure, never miss the opportunity to insist on this. Your colleagues are immense assets, turn to them, nobody ever knows everything, it is no shame. Even the most intelligent do forget at times. Patient's today claim they know more than doctors. Intellectual humility only makes you grow intellectually, morally and to gain respect from your patients and peers. Always have in the back of your mind “who can better handle this patient”, I mean, the spirit of referral. In the first place, the welfare of the patient, not your pride, is the key justification of medical practice. Patients are increasingly more demanding, a gradual transition from medical paternalism to “patient Paternalism”. It can be very frustrating at times, for sure. You shall respect the wishes of your patient, but you remain the doctor. Your biggest enemies shall be the politicians. You know, they really don't care much about you, so avoid the headache.

You shall be overworked and not receive the salary you expect or deserve. Be prepared to appear in court some time in your career, that s life. This must not scare you, but should make you practice evidence based medicine, with highest levels of moral- ethical standards. Your key headache shall be too much information. Be careful with tomorrow's quest for the so called evidence in medicine. Read them, but never be carried away by conclusions from clinical trials, systematic reviews and Meta- analysis other forms of complicated clinical research. You know what, most are incentive driven and at times just pure lies. It's true that most of your time shall be consecrated to secretarial duties filling forms here and there, being on computers, rather than on an actual empathic exchange with your patient. You will have to fight against this. Every patient shall be unique. The clock must tick in your mind now, more than ever before that statistical significance has nothing to do with clinical significance, as well as clinical significance must never dictate holistically how you should handle your patient. Every patient is unique. You shall actively listen to your patient for that might be the only thing she needs. Allow patients to speak, please, you might be the only person she has talked to for a long time. The world shall ask from you far more than what you can offer, but just do the best you can. Indeed, if you can take a good medical history and examine your patient with respect, you did your job. Forget complicated laboratory investigations, radiological interventions, they do help at times, but diluted the very meaning of our honorable art. They must only guide you in your decision making process. It shall be challenging, very challenging, but you are up to the task. Read your books and nice day.

